# The use of active learning strategies in healthcare colleges in the Middle East

**DOI:** 10.1186/s12909-019-1580-4

**Published:** 2019-05-14

**Authors:** Yazed AlRuthia, Solaiman Alhawas, Faris Alodaibi, Lama Almutairi, Reem Algasem, Haitham K. Alrabiah, Ibrahim Sales, Hana Alsobayel, Yazeed Ghawaa

**Affiliations:** 1 Department of Clinical Pharmacy, College of Pharmacy, King Saud University, P.O. Box 2454, Riyadh, 11451 Saudi Arabia; 2 Department of Pharmaceutical Care, King Faisal Specialist Hospital and Research Center, Riyadh, Saudi Arabia; 3 Department of Rehabilitation Sciences, College of Applied Medical Sciences, King Saudi University, Riyadh, Saudi Arabia; 4 King Abdulaziz University Hospital, Riyadh, Saudi Arabia; 5 Department of Medicinal Chemistry, College of Pharmacy, King Saud University, Riyadh, Saudi Arabia

**Keywords:** Active learning, Healthcare education, Middle East

## Abstract

**Background:**

Multiple studies have explored the use of active learning strategies among faculty members in different healthcare colleges worldwide, however, very few have described the use of these strategies in the Middle East. The aim of this study was to evaluate the extent of the implementation of active learning and its various techniques across different fields of healthcare education in various countries in the Middle East.

**Methods:**

A Web-based questionnaire was developed to obtain information on the use of active learning methods. This survey was disseminated among faculty members in healthcare colleges in 17 Middle Eastern countries.

**Results:**

Out of 22,734 online invitations that were sent to faculty members in different healthcare colleges, 2085 (9.17%) accepted the invitations, however, only 722 (34.63%) of those who agreed to participate filled out the questionnaire. Eighty-seven percent of the responders utilized at least one technique of active learning. Active learning was used more frequently by female responders. For example, 54.30% of the female responders reported using learning by teaching as one of their teaching methods compared to 41.30% of their male counterparts (*p* = 0.0005). The various forms of active learning were used at similar levels in both public and private healthcare colleges. Only minor differences were seen among different age groups or academic positions of the responders, but significant variabilities were noted among the several fields of healthcare education. For example, 61.54% of responders from the nursing faculty reported using reaction to videos as one of their teaching methods compared to 31.11% of their counterparts in the faculty of dentistry (*p* = 0.0021). The most frequently reported obstacles interfering with the effectuation of active learning include the lack of technical support and time constraints.

**Conclusions:**

Although some barriers to the implementation of active learning exist, it is extensively used by faculty members in healthcare colleges in the Middle East.

**Electronic supplementary material:**

The online version of this article (10.1186/s12909-019-1580-4) contains supplementary material, which is available to authorized users.

## Background

Recent decades witnessed an increase in the efforts to enhance the traditional methods of teaching, including those utilized in institutions of higher education [1, 2]. This search for novel approaches embraced the active learning technique, which comprises instructional methods that compel the student to become thoroughly engaged in the process of gaining knowledge [3, 4]. Unlike the traditional learning, active learning requires the students to take part in activities that involve higher-order thinking [4, 5]. It shifts the control of the process from the teacher to the students and helps students to take responsibility for their own learning [4]. Evidence on the higher efficiency of active learning over the conventional methods is growing [6–8]. A meta-analysis of data obtained from 225 studies revealed that active learning in undergraduate science courses resulted in a 6% improvement in student performance on exams and that students participating in courses taught by traditional lectures were 50% more likely to fail [9]. Given this shift in teaching methods, the new standards of the Accreditation Council for Pharmacy Education (ACPE) recommend the inclusion of the principles of active learning in the curricula to promote student learning and achievement of professional abilities, such as critical thinking, communication, and self-learning [5, 10, 11].

A variety of active learning techniques have been described in the literature. The most common classroom-based active learning strategies include cooperative learning, problem-based learning, case-based learning, and ability-based education [12–16]. Although active learning has gained enthusiastic supporters among innovation-embracing faculty, several faculty members regard it as a transient fashion [3]. Additionally, many obstacles negatively affect the implementation of active learning techniques. They include an inadequate time to cover the content of a course, excessive pre-class preparation requirements, difficulties in implementation in a large-class setting, lack of resources to support active learning, perception by some faculty that they are good lecturers, and avoidance of risk associated with switching to a different teaching paradigm [17].

Several studies have demonstrated the acceptability and the increasing use of active learning principles in the education of healthcare professionals in the Middle East [18, 19]. Recently, six international Doctor of Pharmacy degree programs were certified by the ACPE, and four of them were in the Middle East [20]. That decision indicates that these programs are aligning their curricula with the ACPE standards. Numerous studies in the field of healthcare education, performed specifically in the Middle East, have evaluated the efficiency of active learning in comparison with traditional lecturing [14, 15, 21]. However, the extent of utilization of active learning methods across different healthcare specialties in the Middle East is yet to be explored. Thus, the objective of the present study was to assess the use of active learning methods in healthcare programs in various Middle Eastern countries as well as to explore the difficulties that might be hindering the adoption of these teaching strategies among faculty members in different healthcare programs in this region.

## Methods

A new Web-based questionnaire was developed to obtain information regarding the most commonly employed active learning methods, potential difficulties in their implementation, the sociodemographic characteristics and academic positions of the faculty members. Eight questions were asked: 1. Age; 2. Gender; 3. College (medicine, pharmacy, dentistry, applied medical sciences, nursing, public health, other); 4. Academic rank (teaching assistant, lecturer, assistant professor, associate professor, professor); 5. Country; 6. Source of funding for the academic institution (public, private); 7. Active learning methods (class discussion, think-pair-share, learning cell, collaborative learning, student debate, reaction to videos, small group discussion, class game, learning by teaching, gallery walk, case studies and problem-based learning, flipped classroom, gamification, and computer-based learning); and 8. Reasons for not adopting active learning strategies (lack of technical support, lack of administrative support, no appreciation, time constraint, and disinterest). Multiple answers were possible for questions 7 and 8 [Additional file 1].

The face and content validity of the questionnaire were independently checked by three faculty members from the colleges of applied medical sciences, medicine, and pharmacy. This survey was disseminated among various healthcare colleges in the Middle East. These institutions were identified by visiting the websites of the ministries of education or higher education of each country, and by manually searching for active websites of healthcare colleges in 17 Middle Eastern countries. Thereafter, a list of email addresses for faculty members in different healthcare colleges in each of the 17 Middle Eastern countries (Saudi Arabia, Qatar, Egypt, Israel, Iraq, Turkey, Bahrain, United Arab Emirates, Sudan, Oman, Jordan, Palestine, Yemen, Iran, Lebanon, Cyprus, and Tunisia) was prepared. The corresponding author was then asked to email a cover letter to all identified email addresses describing the purpose of this online survey as well as an invitation to participate in the study by filling out an online questionnaire that will be sent automatically should they agree to participate in the study.

The reliability of the questionnaire was assessed using the Cronbach’s alpha method [22]; the test returned an alpha value of 0.70 indicating a satisfactory level of reliability. The sociodemographic and academic institutions’ characteristics of the responders (age, gender, college, academic rank, country, and source of institution funding) were presented as frequencies and percentages. Chi-square and Fisher’s exact tests were performed, as appropriate, to compare the proportions of responders who used each of the listed active learning methods (class discussion, think-pair-share, learning cell, collaborative learning, student debate, reaction to videos, small group discussion, class game, learning by teaching, gallery walk, case studies and problem-based learning, flipped classroom, gamification, and computer-based learning) across gender, age groups, academic ranks, and healthcare colleges. Also, Chi-square test was used to compare the proportions of responders who reported each of the listed reasons for not adopting active learning strategies (lack of technical support, lack of administrative support, no appreciation, time constraint, and disinterest) across gender and source of funding for the academic institution. Statistical analysis was performed using Statistical Analysis Software version 9.2 (SAS Institute, Inc., Cary, NC, USA). *p* values of less than 0.05 were considered to be statistically significant.

## Results

**Fig. 1:**
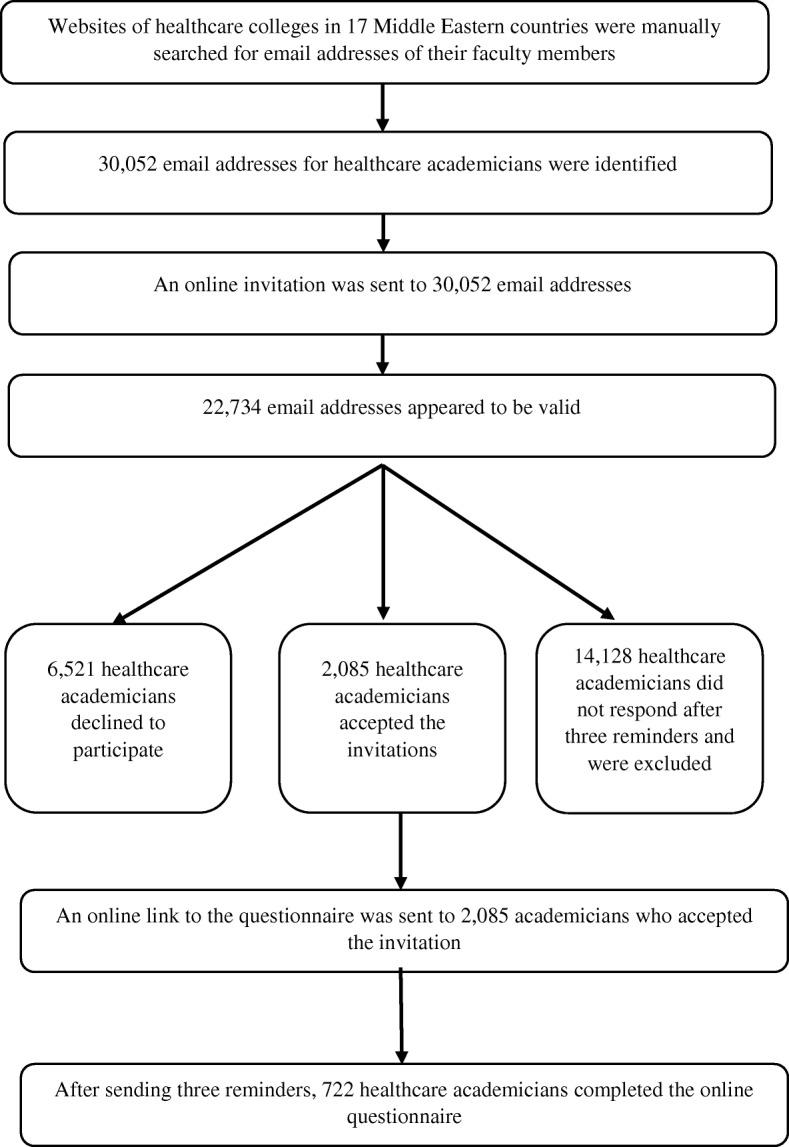
The flow diagram of recruitment of study responders

The search for contact information of faculty members in healthcare educational institutions yielded valid email addresses of 22,734 academicians, of which 722 responded to the provided questionnaire (Fig. 1). Most of the responders were 44 years of age or younger (67%), worked in pharmacy or medical colleges (55%), hold positions of lecturer or assistant professor (63%), and were employed in public institutions (85%). Responses were obtained from academicians representing all 17 Middle Eastern countries, although a disproportionality in the number of replies between different countries was noted (Table 1).

**Table 1:** Sociodemographic characteristics of the responders

Characteristic	Number of responders n (%)
Gender	
Male	385 (53.32)
Female	337 (46.68)
Age (years)	
20–24	4 (0.55)
25–34	205 (28.39)
35–44	274 (37.95)
45–54	141 (19.53)
55–64	79 (10.94)
65–74	17 (2.35)
75–84	2 (0.28)
College	
Dentistry	90 (12.47)
Applied medical sciences	138 (19.11)
School of public health	15 (2.08)
Pharmacy	202 (27.98)
Medicine	195 (27.01)
Nursing	78 (10.8)
Other^a^	4 (0.55)
Rank	
Teaching assistant	93 (12.88)
Lecturer	231 (31.99)
Assistant professor	224 (31.02)
Associate professor	86 (11.91)
Professor	88 (12.19)
Country	
Kingdom of Saudi Arabia	410 (56.79)
Qatar	4 (1.25)
Egypt	143 (19.81)
Israel	23 (3.19)
Iraq	57 (7.89)
Turkey	18 (2.49)
Bahrain	12 (1.66)
United Arab Emirates	13 (1.8)
Sudan	6 (0.83)
Oman	13 (1.8)
Jordan	6 (0.83)
Palestine	5 (0.69)
Other ^b^	7 (0.97)
Institution Funding	
Public	617 (85.46)
Private	105 (14.54)

^a^Child development, preparatory health, student, university-affiliated teaching hospital

^b^Yemen, Iran, Cyprus, Lebanon, Tunisia

**Fig. 2:**
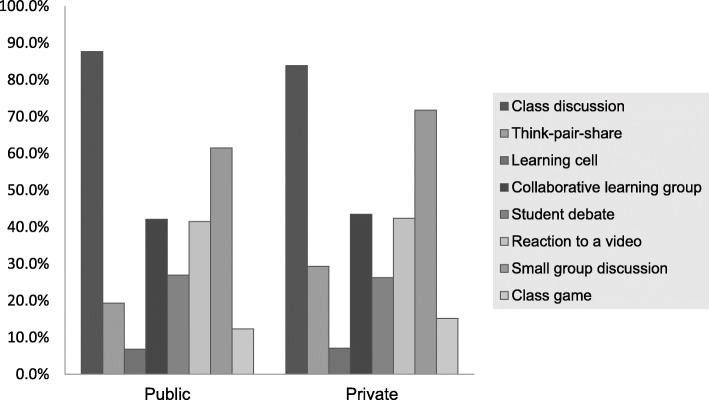
The use of different active learning strategies based on the institution’s funding

The most frequently utilized forms of active learning were by far discussions (Table 2). Class discussion was reported by 87% of the faculty, and small group discussion by 63%. The least used approaches were gallery walk (2.1%), gamification (2.8%) and learning cell (7.1%). With two exceptions (learning cell and student debate), a higher fraction of female responders was engaged in each type of activity, and in most cases, this difference reached statistical significance (*p* < 0.05). For example, the percentage of female responders who reported using class games in the curriculum was three times higher than their male counterparts (20.18% vs. 6.75%; *p* < 0.0001). Moreover, the percentage of female responders who reported using reaction to videos as part of their courses was 1.5 times higher than their male counterparts (50.15% vs. 34.03%; *p* < 0.0001).Of note, the various forms of active learning were employed at a similar frequency in public and private schools (*p* ≥ 0.05) (Fig. 2).

**Table 2:** The use of active learning methods among health care academicians in the Middle East

Active learning method	Responder	Gender		*p*-value
		Male	Female	
	n (%)	n (%)	n (%)	
Class discussion	630 (87.26)	323 (83.90)	307 (91.10)	0.0038^*^
Think-pair-share	149 (20.64)	68 (17.66)	81 (24.04)	0.0348^*^
Learning cell	51 (7.06)	31 (8.05)	20 (5.93)	0.2680
Collaborative learning	307 (42.52)	150 (38.96)	157 (46.59)	0.0386^*^
Student debate	196 (27.15)	109 (28.31)	87 (25.82)	0.4519
Reaction to videos	300 (41.55)	131 (34.03)	169 (50.15)	< 0.0001^*^
Small group discussion	455 (63.02)	229 (59.48)	226 (67.06)	0.0353^*^
Class game	94 (13.02)	26 (6.75)	68 (20.18)	< 0.0001^*^
Learning by teaching	342 (47.37)	159 (41.30)	183 (54.30)	0.0005^*^
Gallery walk	15 (2.08)	6 (1.56)	9 (2.67)	0.2959
Buzz group and brainstorming	193 (26.73)	90 (23.38)	103 (30.56)	0.0295^*^
Case studies and problem based learning	382 (52.91)	186 (48.31)	196 (58.16)	0.0082^*^
Flipped classroom	73 (10.11)	33 (8.57)	40 (11.87)	0.1425
Gamification	20 (2.77)	7 (1.82)	13 (3.86)	0.0957
Computer-based learning	255 (35.32)	124 (32.21)	131 (38.87)	0.0616

^*^Indicates significant difference between males and females (*p <* 0.05)

Some differences in the incidence of implementation of active learning were seen among faculty members of different ages (Table 3). For example, the percentage of responders between 25 to 34 years of age who reported applying collaborative learning was 31.22% compared to 46.18 and 52.94% among those between 45 to 54 and 65 to 74 years of age, respectively, (*p* = 0.0054). Furthermore, responders between 25 to 34 years of age reported using reaction to videos more often than their counterparts between 65 to 74 years of age (45.85% vs. 29.41%; *p* = 0.0178). On the other hand, responders aged between 65 to 74 years reported using case studies and problem based learning, flipped classroom, and gamification more often than their counterparts in other age groups (*p* < 0.05).

**Table 3:** The use of active learning methods among different age groups of healthcare academicians in the Middle East

Active learning method	Age group (years)							*p*-value
	20–24 (4 resp.) n (%)	25–34 (205 resp.) n (%)	35–44 (274 resp.) n (%)	45–54 (141 resp.) n (%)	55–64 (79 resp.) n (%)	65–74 (17 resp.) n (%)	75–84 (2 resp.) n (%)	
Class discussion	3 (75.00)	177(86.34)	247(90.15)	125(88.65)	63(79.75)	13(76.47)	2 (100)	0.1693
Think-pair-share	2 (50.00)	31 (15.12)	64 (23.36)	32 (22.70)	16(20.25)	4 (23.53)	0 (0.00)	0.2288
Learning cell	1 (25.00)	7 (3.41)	20 (7.30)	16 (11.35)	5 (6.33)	2 (11.76)	0 (0.00)	0.0924
Collaborative learning	3 (75.00)	64 (31.22)	129(47.08)	66 (46.81)	36(45.57)	9 (52.94)	0 (0.00)	0.0054^*^
Student debate	1 (25.00)	42 (20.49)	79 (28.83)	44 (31.21)	25(31.65)	5 (29.41)	0 (0.00)	0.2553
Reaction to videos	3 (75.00)	94 (45.85)	125(45.62)	51 (36.17)	21(26.58)	5 (29.41)	1 (50.00)	0.0178^*^
Small group discussion	4(100.00)	112(54.63)	182(66.42)	92 (65.25)	52(65.82)	11(64.71)	2(100.00)	0.0701
Class game	0 (0.00)	25 (12.20)	44 (16.06)	16 (11.35)	6 (7.59)	3 (17.65)	0 (0.00)	0.4257
Learning by teaching	4(100.00)	91 (44.39)	127(46.35)	74 (52.48)	39(49.37)	7 (41.18)	0 (0.00)	0.1761
Gallery walk	0 (0.00)	5 (2.44)	4 (1.46)	4 (2.84)	1 (1.27)	1 (5.88)	0 (0.00)	0.8527
Buzz group and brainstorming	2 (50.00)	38 (18.54)	79 (28.83)	46 (32.62)	22(27.85)	6 (35.29)	0 (0.00)	0.0486^*^
Case studies and problem based learning	2 (50.00)	92 (44.88)	162(59.12)	67 (47.52)	48(60.76)	10(58.82)	1 (50.00)	0.0369^*^
Flipped classroom	0 (0.00)	10 (4.88)	28 (10.22)	19 (13.48)	12(15.19)	4 (23.53)	0 (0.00)	0.0273^*^
Gamification	0 (0.00)	6 (2.93)	6 (2.19)	4 (2.83)	1 (1.27)	3 (17.65)	0 (0.00)	0.0190^*^
Computer-based learning	1 (25.00)	64 (31.22)	114(41.61)	44 (31.21)	28(35.44)	3 (17.65)	1 (50.00)	0.1250

*resp.* responders, *N/A* not applicable

^*^Indicates significant difference among the age groups (*p <* 0.05)

Similar trends were seen when the academic rank of responders was considered (Table 4). The frequency of use of eight types of activities exhibited variability with the type of appointment. For example, the percentage of responders who reported using class discussion was the highest among associate professors (93.02%) compared to 80.68 and 90.18% among professors and assistant professors, respectively, (*p* = 0.0027). On the other hand, professors reported the highest rate of using student debate (36.36%) compared to 27.91 and 29.91% among associate professors and assistant professors, respectively, (*p* = 0.0003). However, assistant professors reported the highest rate of using collaborative learning (46.88%), reaction to videos (45.98%), small group discussion (66.96%), class game (18.30%), and flipped classroom (12.79%) compared to their counterparts from other academic ranks (*p* < 0.05).

**Table 4:** The use of active learning methods across the academic ranks in healthcare colleges in the Middle East

Active learning method	Academic rank					*p*-value
	Teaching assistant (93 resp.) n (%)	Lecturer (231 resp.) n (%)	Assistant professor (224 resp.) n (%)	Associate professor (86 resp.) n (%)	Professor (88 resp.) n (%)	
Class discussion	72 (77.42)	205(88.74)	202 (90.18)	80 (93.02)	71(80.68)	0.0027^*^
Think-pair-share	11 (11.83)	54 (23.38)	53 (23.66)	17 (19.77)	14(15.91)	0.0932
Learning cell	6 (6.45)	9 (3.90)	21 (9.38)	6 (6.98)	9 (10.23)	0.1498
Collaborative learning	23 (24.73)	104(45.02)	105 (46.88)	36 (41.86)	39(44.32)	0.0059^*^
Student debate	8 (8.60)	65 (28.14)	67 (29.91)	24 (27.91)	32(36.36)	0.0003^*^
Reaction to videos	36 (38.71)	108(45.75)	103 (45.98)	36 (41.86)	17(19.32)	0.0002^*^
Small group discussion	44 (47.31)	151(65.37)	150 (66.96)	52 (60.47)	58(65.91)	0.0143^*^
Class game	9 (9.68)	32 (13.85)	41 (18.30)	5 (5.81)	7 (7.95)	0.0139^*^
Learning by teaching	41 (44.09)	103(44.59)	111 (49.55)	46 (53.49)	41(46.59)	0.5815
Gallery walk	2 (2.15)	5 (2.16)	5 (2.23)	0 (0)	3 (3.41)	0.6217
Buzz group and brainstorming	18 (19.35)	61 (26.41)	67 (29.91)	18 (20.93)	29(32.95)	0.1375
Case studies and problem based learning	42 (45.16)	121(52.38)	130 (58.04)	39 (45.35)	50(56.82)	0.1286
Flipped classroom	1 (1.08)	21 (9.09)	31 (13.84)	11 (12.79)	9 (10.23)	0.0127^*^
Gamification	4 (4.30)	2 (0.87)	9 (4.02)	1 (1.16)	4 (4.55)	0.1323
Computer-based learning	22 (23.66)	88 (38.10)	91 (40.63)	22 (25.58)	32 (36.36)	0.0129^*^

*resp.* responders

^*^Indicates significant difference between different academic ranks (*p <* 0.05)

Nine of 15 types of active learning activities showed significant variability among schools in different fields of healthcare education (Table 5). Responders from public health schools reported the highest utilization rates of collaborative learning (80%), class game (46.67%), buzz group and brainstorming (46.67%), and flipped classroom (33.33%) compared to their counterparts from other healthcare colleges (*p* < 0.05). On the other hand, responders from nursing schools reported the highest utilization rates of think-pair-share (33.33%) and reaction to videos (61.54%) compared to their counterparts from other healthcare colleges (*p* < 0.05). However, responders from the colleges of applied medical sciences, dentistry, and medicine reported the highest utilization rates of class discussion (93.48%), case studies and problem based learning (63.33%), and small group discussion (69.23%), respectively, compared to their counterparts from other healthcare colleges (*p* < 0.05).

**Table 5:** The use of active learning methods in various types of healthcare colleges in the Middle East

Active learning method	Type of healthcare college						*p*-value
	Pharmacy (202 resp.) n (%)	Medicine (195 resp.) n (%)	Dentistry (90 resp.) n (%)	Nursing (78 resp.) n (%)	Applied medical sciences (138 resp.) n (%)	Public health (15 resp.) n (%)	
Class discussion	177(87.62)	162(83.08)	78(86.67)	70(89.74)	129 (93.48)	12 (80.00)	0.0290^*^
Think-pair-share	37 (18.32)	37 (18.97)	13(14.44)	26(33.33)	30 (21.74)	4 (26.67)	0.0383^*^
Learning cell	13 (6.44)	11 (5.64)	5 (5.56)	6 (7.69)	14 (10.14)	1 (0.67)	0.5386
Collaborative learning	75 (37.13)	75 (38.46)	34(37.78)	34(55.13)	67 (48.55)	12 (80.00)	0.0020^*^
Student debate	57 (28.22)	44 (22.56)	21(23.33)	23(29.49)	46 (33.33)	4 (26.67)	0.4521
Reaction to videos	82 (40.59)	71 (36.41)	28(31.11)	48(61.54)	63 (45.65)	7 (46.67)	0.0021^*^
Small group discussion	115(56.93)	135(69.23)	59(65.56)	49(62.82)	90 (65.22)	6 (40.00)	0.0452^*^
Class game	21 (10.40)	15 (7.69)	10(11.11)	16(20.51)	24 (17.39)	7 (46.67)	< 0.0001^*^
Learning by teaching	100(49.50)	90 (46.15)	32(35.56)	44(56.41)	66 (47.83)	9 (60.00)	0.1309
Gallery walk	5 (2.48)	3 (1.54)	0 (0)	5 (6.41)	2 (1.45)	0 (0.00)	0.1160
Buzz group and brainstorming	41 (20.30)	53 (27.18)	14(15.56)	35(44.87)	42 (30.43)	7 (46.67)	0.0001^*^
Case studies and problem based learning	83 (41.09)	111(56.92)	57(63.33)	43(55.13)	79 (57.25)	7 (46.67)	0.0063^*^
Flipped classroom	12 (5.94)	28 (14.36)	4 (4.44)	8 (10.26)	16 (11.59)	5 (33.33)	0.0022^*^
Gamification	8 (3.96)	4 (2.05)	0 (0.00)	4 (5.13)	4 (2.90)	0 (0.00)	0.4054
Computer-based learning	66 (32.67)	61 (31.28)	33(36.67)	30(38.46)	54 (39.13)	8 (53.33)	0.2249

*resp.* responders

^*^Indicates significant difference between different types of colleges (*p <* 0.05)

The most frequently claimed reasons for not adopting active learning strategies was the lack of technical support and time constraints (Table 6). There was no significant difference between genders in the evaluation of these obstacles, with the only exception of women citing the existence of time constraints more often than men (51.93% vs. 35.84%; *p* < 0.0001). Conversely, several differences were identified when public and private colleges were compared. The faculty in public schools pointed more often than their counterparts in private schools to the lack of technical support (41.17% vs. 25.25%; *p* = 0.0057), lack of administrative support (24.47% vs. 12.12%; *p* = 0.023), and the presence of time constraints (45.22% vs. 30.30%; *p* = 0.0107).

**Table 6:** Reasons for not adopting active learning methods across genders and types of funding in healthcare colleges in the Middle East

Reason	Gender				Source of funding			
	Both genders (722 resp.) n (%)	Male (385 resp.) n (%)	Female (337 resp.) n (%)	*p*-value	Any source (716 resp.)^a^ n (%)	Public (617 resp.) n (%)	Private (99 resp.) n (%)	*p*-value
Lack of technical support	280 (38.78)	156 (40.52)	124 (36.80)	0.3055	279 (38.97)	254 (41.17)	25 (25.25)	0.0057^*^
Lack of administrative support	164 (22.71)	91 (23.64	73 (21.66)	0.5275	163 (22.77)	151 (24.47)	12 (12.12)	0.0230^*^
No appreciation	134 (18.56)	69 (17.92)	65 (19.29)	0.6377	133 (18.58)	122 (19.77)	11 (11.11)	0.1195
Time constraint	313 (43.35)	138 (35.84)	175 (51.93)	< 0.0001^*^	309 (43.16)	279 (45.22)	30 (30.30)	0.0107^*^
Disinterest	110 (15.24)	58 (15.06)	52 (15.43)	0.8916	109 (15.22)	91 (14.75)	18 (18.18)	0.6743
Refrained from answering the question	34 (4.71)	17 (4.42)	17 (5.04)	0.6906	34 (4.75)	27 (4.38)	7 (7.07)	0.4318

*resp.* responders

^*^Indicates significant difference between males and females, or public and private schools (*p <* 0.05)

^a^6 responders did not provide information regarding the type of funding

## Discussion

The current study represents, to the best of our knowledge, the first effort to explore the prevalence of utilization of active learning strategies in the Middle East. The responders were from different Middle Eastern countries and reflected most healthcare specialties. The results of the survey indicated that the active learning methods are prominently applied in this part of the world. Class and small-group discussions were the most commonly used methods by responders of both sexes and of all ages and academic ranks. However, detailed analysis revealed the presence of several substantial differences in how the various techniques are used in the healthcare education system in the Middle East. The major obstacles in the implementation of active learning were identified as well.

It does not come as a surprise that the most frequently utilized forms of active learning were class and small group discussions. Discussion as a form of gaining knowledge was practiced by Socrates in ancient Greece [23], long before the concept of active learning was formally introduced [24–26]. Moreover, this method of active learning does not require any a priori preparation. In fact, approaches requiring significant effort to develop, such as gallery walk or gamification were used by less than 3% of the faculty.

One of the most striking findings of the present research was the fact that female responders were more likely to employ active learning strategies than their male counterparts. The reason for this gender disparity is unknown. Switching to novel teaching techniques may be associated with risk-taking, and it has been extensively documented in a number of observational and experimental studies that women are characterized by a lower propensity to take risks [27, 28]. One potential explanation for the higher participation of women in providing active learning environment may be offered by results of a study which indicated that women tend to adopt more democratic and participative style in their leadership role than men, who tend to prefer more autocratic and directive style [29]. This trait may prevail over the avoidance of risk associated with the introduction of novel teaching methods, resulting in the observed gender difference. Interestingly, a recent study has shown that female students perceive the utilization of active learning approaches as a factor positively contributing to their academic experience more often than their male counterparts [30].

When the frequency of use of active learning was compared between public and private colleges, it became apparent that the type of institutional funding does not have a large impact on the choice of learning methods used. The only exception noted was with the think-pair-share which was utilized to a greater extent than the student debates in private schools as opposed to public schools. Possible reasons for this difference may include smaller class sizes and lower time constraints in private universities.

Our data did not reveal a robust correlation between the age of healthcare faculty members and the use of active learning methods. If the data from the oldest (75–84 years, 2 responders) and the youngest (20–24 years, 4 responders) groups are excluded on the basis of the very low number of responses, the remaining oldest group (65–74 years) had the highest rate of utilization of think-pair-share, learning cell, collaborative learning, class game, gallery walk, buzz group and brainstorming, flipped classroom, and gamification. Thus, the majority (8 of 15) of active learning techniques were used most frequently in this group of seasoned lecturers. This data appears to contradict the notion that the older healthcare faculty members are discouraged from implementing active learning methods by approaching retirement age, or by the self-perception of being a good lecturer after years of teaching [31]. In a European study, younger teachers described old cadre as real obstacles to using active learning methods, claiming that older teachers are cynical, burned out, and do not have the energy to apply new ways of teaching [32]. The current data contradict these sentiments, particularly taking into account that in most European countries faculty must retire at the age of 65, before even reaching the age of responders in the two oldest groups of the present analysis of Middle Eastern countries.

The reason for these major discrepancies remains to be explained. It may reflect the different attitudes toward older members of the society in various parts of the world [33]. However, the alternative explanation in which old professors who are not interested in active learning might have elected not to participate in this study cannot be excluded.

Among the most frequently cited reasons for not adopting active learning methods was time constraint. This finding is consistent with the previously published data [31]. The current results indicate additionally that the female faculty were 45% more likely than their male counterparts to indicate time constraint as a barrier to the implementation of active learning. The underlying cause of this gender-dependent perception remains to be identified. Lack of technical and administrative support as well as time constraints were reported more often by responders from public schools; this might be related to a higher level of bureaucracy in public schools. Alternatively, this may reflect a greater effort of private schools to attract students, who are generally more enthusiastic about active learning than traditional teaching [31, 34]. It is noteworthy, however, that in spite of a smaller number of obstacles in private schools, the extent to which active learning was incorporated in the curriculum was comparable to that seen in public schools (Fig. 2).

### Study limitations

Although this study is the first, to the best of our knowledge, to explore the prevalence of active learning methods utilization in different healthcare colleges in the Middle East, it has multiple limitations. First and foremost, the low response rate (722 responses out of 22,734 sent emails) despite three online reminders that were sent asking faculty members in different Middle Eastern healthcare colleges to participate; this can be due to a multitude of factors. These factors can be cultural in which people in the Middle East are not used to fill out online questionnaires; something that was confirmed in a study that was conducted in Saudi Arabia to compare the response rates between Web and telephone surveys, and found a significantly lower response rate for Web survey compared to telephone survey despite the fact that the number of Internet users is significantly higher than the number of telephone landline subscribers [35]. In addition to the cultural factors, Web surveys had traditionally lower response rates compared to telephone, paper, or personal interview surveys mainly due to people’s concern with the confidentiality of the provided information as well as the reliability of the sources of Web surveys [36, 37]. We have tried to address these issues by using an institutional email with the name and contact information of the corresponding author as well as by sending a cover letter that described the purpose of the study and invited the addressees to participate prior to emailing them the link to the online questionnaire, which was only sent to those who agreed to participate. Another potential factor that may have contributed to the low response rate is the language of the administered online questionnaire [38]. Although the questionnaire was administered in English, which is the language used in teaching in all of the surveyed Middle Eastern healthcare colleges, English is not the native language in any of the surveyed countries. Moreover, the majority of the responders were between 25 to 54 years of age which could be attributable to the low digital literacy level and use of Internet among old faculty members compared to their younger counterparts [39]. In addition, more than 50% of the responders were from Saudi Arabia, which can be due to the familiarity of the responders with the academic institution in which the questionnaire was developed and sent from as well as the higher rate of Internet utilization compared to most Middle Eastern countries [35]. Furthermore, some Middle Eastern countries such as Syria and Morocco were excluded mainly because of the language barrier where most of their healthcare colleges’ curricula are not taught in English. Finally, the use of convenience sampling which was employed due to resource and time constraints is another limitation that may affect the generalizability of the study’s findings.

## Conclusion

Middle Eastern healthcare education policymakers should incentivize the use of active learning strategies among their healthcare faculty members to improve the educational outcomes. Although it might be premature to generalize the findings, the accumulated data provide useful insights regarding the various factors that influence the implementation of active learning strategies in the Middle East. Moreover, it indicates the difficulties that hinder the adoption of novel teaching techniques by healthcare academicians in this part of the world.

## Additional file

Additional file 1: The use of active learning strategies in healthcare colleges in the Middle East questionnaire. (DOCX 16 kb)

## Abbreviation

ACPE: Accreditation Council for Pharmacy Education
