# Supplementary material for: The use of active learning strategies in healthcare colleges in the Middle East
Source: BMC Med Educ. 2019 May 14;19:143. doi: 10.1186/s12909-019-1580-4 (PMC6518770; doi:10.1186/s12909-019-1580-4)
Supplement: Supplementary file 1 — The use of active learning strategies in healthcare colleges in the Middle East questionnaire. (DOCX 16 kb) [file 12909_2019_1580_MOESM1_ESM.docx]

**The Use of Active Learning Strategies in Healthcare Colleges in the Middle East Questionnaire**

1. **Which age group do you belong to?**
   - 25-34 years.
   - 35-44 years.
   - 45-54 years.
   - 55-64 years.
   - 65-74 years.
   - 75-84 years.
   - Other:_________________
2. **Are you:**
   - Male.
   - Female.
3. **Which health care college do you teach in?**
   - College of Medicine.
   - College of Dentistry.
   - College of Pharmacy.
   - College of Applied Medical Sciences (e.g., physiotherapy, health education, nutrition, etc....).
   - College of Nursing.
   - College of Public Health.
   - Other:____________________
4. **What is your academic rank?**
   - Professor.
   - Associate Professor.
   - Assistant Professor.
   - Lecturer.
   - Teaching Assistant.
   - Other:___________________
5. **Which country in the Middle East your academic institution is located in?**
   - Egypt.
   - Iran.
   - Turkey.
   - Iraq.
   - Saudi Arabia.
   - Yemen.
   - Syria.
   - United Arab Emirates.
   - Israel.
   - Jordan.
   - Palestine.
   - Lebanon.
   - Oman.
   - Qatar.
   - Bahrain.
   - Cyprus.
   - Morocco.
   - Algeria.
   - Libya.
   - Tunisia.
   - Mauritania.
   - Somalia.
   - Sudan.
   - Other:______________
6. **The college/university that you work in is a...........academic institution.**
   - Public.
   - Private.
7. **Which of the below active learning methods have you used in teaching your students? (Please check all the boxes that apply)**
   - Class discussion.
   - Think-pair-share.
   - Learning cell.
   - Collaborative learning group.
   - Student debate.
   - Reaction to a video.
   - Small group discussion (e.g., seminars and tutorials).
   - Class game.
   - Learning by teaching.
   - Gallery walk.
   - Brainstorming and buzz groups.
   - Case studies and problem-based learning (e.g., crossovers, Delphi technique, Role play).
   - Flipped Classroom (Inverting your class).
   - Gamification (e.g., Bingo Game, Crossword Puzzle).
   - Computer-based teaching and learning (e.g., Online quizzes, mark assessments and provide feedbacks, Online interactive course materials, Online discussion forums and bulletin boards for online communications, video-conferencing and live lectures).
   - None of the above.
   - Other:________________________
8. **Reasons for not adopting and implementing active learning methods in your view: (Please check all the boxes that apply)**
   - Technical support is lacking.
   - Administrative support to make the teaching and learning process more effective is lacking.
   - No appreciation whatsoever.
   - The department and the college are very helpful and supportive.
   - I refrain from answering this question.
   - Time constraint.
   - Disinterest in using such methods especially from the students side.
   - Other:_____________________________
